# Editorial: AI-driven healthcare delivery, ageism, and implications for older adults: emerging trends and challenges in public health

**DOI:** 10.3389/fpubh.2025.1755410

**Published:** 2025-12-18

**Authors:** Gul Seckin

**Affiliations:** Department of Sociology, University of North Texas, Denton, TX, United States

**Keywords:** ageism, artificial intelligence, digital health adoption, healthcare delivery, older adults

## Introduction

As societies worldwide face rapidly aging populations, healthcare systems are under increasing pressure to adapt. At the same time, artificial intelligence (AI) is transforming healthcare delivery—affecting how diseases are detected, monitored, and managed. However, integrating AI into health systems raises complex questions for older adults: How inclusive are these technologies? Are they designed with older populations in mind? Do they challenge or reinforce ageist biases?

This Research Topic—*AI-driven healthcare delivery, ageism, and implications for older adults: emerging trends and challenges in public health*—was created to explore these intersections. It gathers seven diverse articles that examine how AI-driven tools are being developed, evaluated, and adopted in settings involving older adults, and how ethics, equity, and ageism are being addressed throughout the process. Together, this Research Topic of work highlights a growing awareness that technological innovation must be paired with human-centered design, inclusive policies, and ethical foresight.

## Framing the issues: innovation, inclusion, and ageism

AI has enormous potential to support healthy aging—providing tools for early detection, managing chronic diseases, rehabilitation, and even companionship through conversational agents. However, this promise is balanced by genuine concerns: digital exclusion, underrepresentation in datasets, age-biased algorithms, and limited health-system readiness for fair deployment.

This Research Topic emphasizes three overarching themes:

1. Adoption and Inclusion—How willing and able are older adults to engage with AI tools? Digital health literacy, perceived value, and usability are key determinants.
2. Clinical and Diagnostic Innovation—AI applications in gait analysis, aphasia therapy, Alzheimer's diagnostics, and chronic disease monitoring are quickly advancing and show promise for older populations.
3. Ethical and Systemic Concerns—Using AI in healthcare prompts essential questions about transparency, accountability, and the ongoing presence of ageism in digital systems.

Each contribution in this Research Topic tackles these issues from a unique perspective, enriching the overall discussion on AI and aging in public health.

## Highlights of the Research Topic

### Technology acceptance and digital health literacy

Wu and Lim examined older adults' willingness to adopt wearable health technologies by combining the UTAUT2 model with the Technology Readiness Index. Their findings show that performance expectancy, effort expectancy, and motivation significantly influence behavioral intention, and that digital health literacy plays a crucial moderating role. The study highlights the need for tailored education and support systems to help older adults engage effectively with AI-enabled wearables. In a related study, Yu and Chen explored older adults' acceptance of healthcare chatbots. They found that trust, ease of use, and perceived usefulness are key factors in acceptance, suggesting that designers should focus on intuitive interfaces and clear communication, especially for populations unfamiliar with AI.

### AI in diagnosis and monitoring

Naseem et al. proposed an AI-based framework using gait sequence and foot-pressure image data to diagnose sarcopenia—an age-related condition that affects mobility and independence. Their model shows the potential for AI to support early intervention strategies for older adults at risk of functional decline. Similarly, Jiang et al. introduced a multi-modal AI framework (ReIU) for Alzheimer's disease assessment. By integrating data from sensors, imaging, and clinical parameters, their system offers a promising approach to managing complex neurodegenerative conditions in aging populations. Zhong's review on AI-assisted assessment and treatment of aphasia further contributes to the diagnostic theme. Aphasia, often linked with stroke and neurodegeneration in older adults, may benefit greatly from AI-powered speech and language tools. However, the review also highlights current gaps in real-world validation and the need for inclusive training datasets.

### System-level perspectives and ethical reflection

Xie et al. examined the impact of China's “Internet Plus” smart healthcare policy on older adults with chronic diseases. Using data from the China Family Panel Studies, their findings show that smart healthcare is linked to better physical and mental health outcomes—providing real-world evidence that policy frameworks can influence digital health equity. Kahraman et al. provided a qualitative exploration of physicians' ethical concerns about AI in medicine. While clinicians recognized the potential of AI, they strongly emphasized that “the final decision should rest with a human.” Concerns about transparency, accountability, and the risk of age-related bias highlight the need for ethical safeguards, especially when AI systems are used in clinical decisions involving vulnerable older populations.

## Broader implications and future directions

The articles in this Research Topic highlight an important shift: from viewing older adults as passive recipients of technology to recognizing them as active participants in the digital health ecosystem. However, significant challenges remain. To ensure AI benefits aging populations fairly, several key areas need to be focused on.

- Human-Centered Design: Co-design with older adults is essential to ensure AI tools are usable, acceptable, and accessible.
- Data Inclusion: AI systems must be trained on diverse and age-representative datasets to avoid perpetuating age-related bias.
- Ethical Governance: Prioritize transparent and accountable AI deployment, especially in high-stakes clinical decision-making.
- Digital Equity Policies: Bridging the digital divide requires investment in infrastructure, training, and systemic preparedness across health systems.
- Interdisciplinary Collaboration: The development and implementation of AI tools should involve not only engineers and clinicians but also medical sociologists, gerontologists, public health experts, and older adults themselves.

## Looking forward

This Research Topic sheds light on the multifaceted relationship between AI and aging, encompassing technological readiness and ethical reflection, as well as diagnostic innovation and systemic equity. Although the articles differ in methods, settings, and focal technologies, several overarching themes emerge. First, they collectively show that AI-powered tools are increasingly integrated into various aspects of older adults' health trajectories, from managing chronic diseases in community settings to specialized diagnostics and rehabilitation. Second, they demonstrate that older adults are both capable and often willing to engage with chatbots, wearables, and smart health platforms when these systems are user-friendly, affordable, and meaningfully incorporated into their lives. Third, the contributions remind us that clinicians' ethical principles and regulatory frameworks remain essential in shaping how AI impacts aging populations. This Research Topic provides a timely and necessary foundation for ongoing inquiry into AI-driven health systems for older adults. Yet, the work is far from finished. Several critical questions remain:

- How do we ensure that AI tools develop in line with the real-world needs and preferences of older users?
- What regulatory framework is needed to protect against algorithmic harms in older adults?
- How can we design interdisciplinary curricula that prepare future healthcare workers to incorporate AI without abandoning the human touch?

As the global population continues to age, the demand for AI-powered health systems that support older adults rather than exclude them becomes more urgent. We hope this Research Topic fosters timely and critical discussion at the intersection of technology, aging, and public health.

